# Unemployment Hysteresis by Sex and Education Attainment in the EU

**DOI:** 10.1007/s13132-023-01106-1

**Published:** 2023-01-17

**Authors:** Juan Carlos Cuestas, Luis Gil-Alana

**Affiliations:** 1 Department of Economics and IEI, Jaume I University, Castellón de La Plana, Spain; 2 Department of Economics and Finance, Tallinn University of Technology, Tallinn, Estonia; 3 Department of Economics, University of Navarra, Pamplona, Spain; 4 Faculty of Law, Business and Government, Universidad Francisco de Vitoria, Madrid, Spain

**Keywords:** Unemployment, Male and female, Education attainment, Fractional integration, C22, F15

## Abstract

In this paper, we analyse the degree of persistence of shocks in unemployment rates for a group of 22 European countries, disaggregating the data by sex and education attainment. By means of using a long-memory model with fractional integration techniques, we find high levels of persistence in the majority of the cases with orders of integration which are around 1. Also, we find that women and those with higher education are the ones which shocks tend to have less lasting effects since they refer to the series with the lowest orders of integration, in some cases being significantly below 1 and thus showing mean reversion. Policy implications are derived from our analysis.

## Introduction

The issue of the analysis of the time series properties of the unemployment rate of different developed countries has traditionally been the subject of numerous studies since the 1980s. However, there is far less empirical analysis on the dynamic properties of unemployment distinguishing by sex and education attainment than for aggregated data. Unemployment is a social stigma that epitomises the health of an economy. Some European countries such as Spain and Greece have never managed to recover the pre-Great Recession unemployment rate levels. After the COVID-19 pandemic, things have only taken a turn for the worse.

In order to analyse the effect of shocks on different groups for the unemployment rate in a set of European countries, we analyse the order of integration of unemployment rates by gender and schooling attainment. The analysis of the order of integration *I*(*d*) is relevant for policy matters. If the variable is integrated of an order less than 0.5, the variable is considered to be stationary process with shocks only lasting temporarily. However, if the order of integration is in the interval [0.5, 1), the variable is a non-stationary process but mean reverting. This means that shocks will have long-lasting effects although the variable will eventually revert to the equilibrium; finally, if the order of integration is 1 or over, shocks will never die out, and policy intervention will be necessary to revert the effects of a shock on the variable.

This empirical testing can be linked to the traditional hypothesis of unemployment: hysteresis, persistence and the natural rate of unemployment or non-accelerating inflation rate of unemployment (NAIRU) (Friedman, 1968; Phelps, 1968). Hence, by testing the order of integration, not only are we testing the effect of shocks on the variable, but also which of the hypotheses is fulfilled by the data. Hysteresis implies that the variable does not revert to the equilibrium after a shock, meaning that the order of integration is 1 or over. If the natural rate of unemployment hypothesis is valid, the order of integration should be less than 1. Finally, the persistence hypothesis establishes that the rate of unemployment needs a long time to revert to the equilibrium after a shock, which is epitomised by an order of integration between 0.5 and 1 (Leon-Ledesma, 2002; Leon-Ledesma & McAdam, 2004; Christopoulos & León‐Ledesma, 2007; Cuestas et al., 2011; Canarella et al., 2019).

In recent years, there has been a significant increase in the number of papers analysing these hypotheses for the unemployment rate (Akdoğan, 2017; Yaya et al., 2021); however, only a few have analysed it by gender (Passinhas & Proença, 2020) and education level (Faďoš & Bohdalová, 2017; Torgovitsky, 2019).^1^ In the case of Passinhas and Proença (2020), the focus is only on the case of Portugal. Faďoš and Bohdalová (2017) focus on an aggregate level for the EU, Spain and Switzerland, \as Torgovitsky (2019) on a methodological contribution analyses the unemployment rate dynamics for the USA though limited to the 2011–2013 period. For this reason, in this paper, we aim to analyse the degree of unemployment persistence over different levels of schooling segregating by gender and focussing on a larger pool of countries from the EU.

The remainder of the paper is organised as follow: the second section briefly describes the main theories dealing with unemployment dynamics, and the third section focuses on the empirical results. The fourth section deals with the discussion and policy recommendations.

## A Brief Description of the Empirical Testing of Unemployment Theories

As mentioned in the previous section, the NAIRU hypothesis implies that there is an unemployment rate of equilibrium at which the variable tends to revert. This implies that the Phillips Curve is vertical, and regardless of the inflation rate, the unemployment rate does not change. Despite this fact, we may observe transitory deviations from the equilibrium. Hence, from an econometric point of view, the unemployment rate should be an *I*(0) process. This is of course, provided that the fundamentals which define the equilibrium rate of unemployment do not change over time (Holmes et al., 2013). More recently, we find several contributions related to our matter; Ghoshray et al. (2016) analyse how structural changes affect the evolution of adult and youth unemployment rates, finding that only adult unemployment is more sensitive to structural breaks due to events, whereas youth one is more sensible to the business cycle. Cairó and Cajner (2018) find that the fact that more educated workers enjoy greater employment stability has to do with the on-the-job training and the formal education itself. Su et al. (2022) find that the COVID-19 has had a significant effect on unemployment for a number of European countries, i.e. France, Germany, Italy, Spain and UK. For this purpose, they use unit root tests with non-linearities. In a seminal contribution, Ahn and Hamilton (2020) establish that changes in the composition of new incomers into unemployment tend to be the most significant ones in economic recessions.

However, if the fundamentals of the equilibrium unemployment rate do change over time, this equilibrium rate may also be time-varying (Phelps, 1994; Layard et al., 2005; Cuestas & Harrison, 2014). In this case, the unemployment rate should be a stationary process around a time trend, which may be linear or non-linear, with structural breaks (Meng et al., 2017).

Testing for the NAIRU hypothesis empirically has traditionally relied on unit root or stationarity tests to label the variables as I(1) or I(0). However, fractional integration methods provide us with a more flexible framework since the parameter *d* may be a non-integer number (see, e.g. Caporale & Gil-Alana, 2008). Fractionally integrated (or *I*(*d*)) models are as follows:

1 $${\left(1-L\right)}^{d}{x}_{t}={u}_{t},\quad t=1,\dots .,T,$$

where *u*_*t*_ is a covariance stationary *I*(0) process, whose spectral density function is positive and finite at the zero frequency, *d* can be any real number, and *L* is the lag operator. We can re-write the above equation by using the expansion:

2 $${\left(1-L\right)}^{d}{x}_{t}={x}_{t}-{dx}_{t-1}+\frac{d\left(d-1\right)}{2}{x}_{t-2}-\frac{d\left(d-1\right)\left(d-2\right)}{6}{x}_{t-3}+\dots$$

provided that

3 $${\left(1-L\right)}^{d}=\sum_{j=0}^{\infty }\left(\begin{array}{c}d\\ j\end{array}\right){\left(-1\right)}^{j}{L}^{j}.$$

Therefore, the closer the parameter *d* is to 1, the more persistent the process is, and the effect of shocks on the variable will last longer. If *d* ∈ (0, 0.5), the series is covariance stationary and mean reverting and therefore the NAIRU hypothesis holds. However, if *d* ∈ [0.5, 1), the series is no longer stationary but still mean reverting, and then we are dealing with the persistence hypothesis. Finally, when *d* ≥ 1, the series is non-stationary and non-mean reverting, and hence, the hysteresis hypothesis is met (Blanchard & Summers, 1987). In the latter, shocks affecting the unemployment rate will never disappear from the behaviour of the variable, and it will not revert to the equilibrium. In Table 1 we summarise these hypotheses.

**Table 1:** Order of integration of unemployment and hypothesis fulfilled

Order of integration	Hypothesis
*d* ∈ *[0,0.5)*	NAIRU or natural rate
*d* ∈ *(0,0.5)* + *trend/structural changes*	NAIRU structuralist view point
*d* ∈ *[0.5,1)* *d* ≥ *1*	Persistence Hysteresis

## Empirical Analysis

The data for this paper are quarterly seasonally adjusted observations for unemployment rates by sex and educational attainment in a group of European countries from 2003:Q2 until 2019:Q4 for the 15 to 74 age group. We have omitted observations in 2020 to avoid the effect of the COVID-19 pandemic. The data was extracted from *Eurostat* on 11 March 2021. The code of the variable is *une_educ_q.* The data are displayed in the Appendix. The different levels of education attainment are as follows: less than primary, primary and lower secondary education (levels 0–2); upper secondary and post-secondary non-tertiary education (levels 3 and 4); and tertiary education (levels 5–8).

The estimated model is as follows:

4 $$y\left(t\right)=a+\beta t+x\left(t\right);\quad {\left(1-L\right)}^{d}x\left(t\right)=u\left(t\right).$$

where *y*(*t*) is the observed time series, *α* and *β* are unknown coefficients on the intercept and the linear time trend, and *x*(*t*) is assumed to be *I*(*d*), with *d* being another parameter to be estimated. The estimation is based on the Whittle function expressed in the frequency domain, and for this purpose, we use a simple version of the tests of Robinson (1994) widely used in empirical applications (see, e.g. Gil-Alana & Robinson, 1997). These tests have the advantage that they do not impose stationarity and thus do not require first differentiation prior to the estimation in case of non-stationary series. In addition, it is the most efficient method in the Pitman against local departures from the null and have an asymptotic standard *N*(0, 1) distribution.

Across the tables, we present the estimates of *d* and the 95% bands of the non-rejection values of *d* using Robinson (1994)’s method in Eq. (4) under three different modelling specifications for the deterministic terms. Thus, in the second column, we present the results supposing that *α* = *β* = *0* a priori, so no deterministic components are included in the model; in the third column, we report the estimated values of *d* under the assumption that *α* is unknown and *β* = *0* a priori, i.e. including an intercept in the regression model; finally, in the last column, we estimate *d* along with *α* and *β* also estimated from the data. We display in the table in bold the most adequate model for each series. This is based on the corresponding *t* values on the estimated coefficients in the *d-*differenced regressions, noting that the joint representation of the two equalities in Eq. (4) produces a new regression model where the errors are *I*(0) by assumption so standard *t* values (*p* values) on the estimated coefficients hold.

In Tables 2, 3 and 4, we show the results for the estimated order of integration for the total unemployment by education attainment. In Table 5, we report a summary of the three tables. From Tables 2, 3, 4 and 5, we find that there is evidence of mean reversion in four countries for the unemployment at low level (Austria, Belgium, Finland and Slovenia); a single country with medium level (Belgium) and another group of four countries with high level of education (Austria, Belgium, Croatia and Denmark). In these cases, the differencing parameter is statistically significantly lower than 1, supporting thus transitory shocks and reversion to the mean. In all the other cases, the estimates of the differencing parameter are equal to or higher than 1 at the 5% level, implying lack of mean reversion. Thus, only for Belgium do we find evidence of mean reversion across the different degrees of schooling. A decreasing trend in Germany is also found for the three groups of education level. This deterministic trend is most likely related to technological progress and policy measures to make the labour markets more flexible. Surprisingly, a positive trend is found for Sweden for low levels of education but not for medium and high education levels.

**Table 2:** Estimates of the differencing parameter: total unemployment (levels 0–2)

Country	No terms	An intercept	An intercept and a linear time trend
Austria	0.89 (0.69, 1.09)	0.67 (0.52, 0.88)*	0.67 (0.50, 0.89)
Belgium	0.77 (0.53, 0.98)	0.47 (0.34, 0.63)*	0.47 (0.34, 0.63)
Bulgaria	0.95 (0.74, 1.18)	**1.20 (1.07, 1.37)**	1.20 (1.07, 1.37)
Croatia	0.89 (0.69, 1.14)	**0.93 (0.76, 1.16)**	0.93 (0.76, 1.16)
Cyprus	0.98 (0.81, 1.18)	**1.02 (0.87, 1.20)**	1.02 (0.87, 1.20)
Czechia	1.06 (0.85, 1.29)	**1.30 (1.10, 1.59)**	1.30 (1.10, 1.59)
Denmark	0.89 (0.68, 1.13)	**0.87 (0.73, 1.06)**	0.87 (0.73, 1.06)
Finland	0.93 (0.74, 1.18)	0.56 (0.44, 0.72)*	0.55 (0.41, 0.72)
France	0.88 (0.61, 1.12)	**0.96 (0.80, 1.17)**	0.96 (0.80, 1.17)
Germany	0.99 (0.67, 1.22)	1.06 (0.93, 1.24)	**1.07 (0.90, 1.25) -**
Greece	1.16 (0.96, 1.36)	**1.35 (1.21, 1.52)**	1.35 (1.21, 1.51)
Hungary–Ireland	1.01 (0.77, 1.19)	**1.15 (1.03, 1.29)**	1.15 (1.03, 1.29)
Ireland	1.26 (1.10, 1.47)	**1.29 (1.15, 1.45)**	1.29 (1.15, 1.45)
Italy	0.83 (0.59, 1.09)	**1.09 (0.95, 1.25)**	1.09 (0.95, 1.25)
Netherlands	1.09 (0.89, 1.31)	**1.28 (1.12, 1.50)**	1.28 (1.12, 1.50)
Poland	0.96 (0.67, 1.21)	**1.08 (0.92, 1.29)**	1.08 (0.92, 1.29)
Portugal	1.12 (0.93, 1.34)	**1.38 (1.25, 1.58)**	1.38 (1.24, 1.58)
Romania–Slovakia	0.97 (0.78, 1.19)	**0.73 (0.47, 1.09)**	0.74 (0.47, 1.09)
Slovakia	0.99 (0.76, 1.21)	**0.93 (0.75, 1.24)**	0.92 (0.68, 1.24)
Slovenia	0.74 (0.57, 0.92)	0.79 (0.65, 0.95)*	0.79 (0.65, 0.95)
Spain	1.16 (0.97, 1.38)	**1.86 (1.69, 2.08)**	1.86 (1.69, 2.08)
Sweden	1.03 (0.86, 1.25)	0.75 (0.57, 1.10)	**0.85 (0.66, 1.10) + **

The values in parenthesis are the 95% confidence bands for the estimates of the differencing parameter. In bold, the selected model for each country according to the deterministic terms. *, evidence of mean reversion at the 5% level. Column 4, + / − positive or negative trend

**Table 3:** Estimates of the differencing parameter: total unemployment (levels 3–4)

Country	No terms	An intercept	An intercept and a linear time trend
Austria	0.93 (0.70, 1.16)	**1.01 (0.83, 1.24)**	1.01 (0.83, 1.24)
Belgium	0.95 (0.72, 1.18)	0.71 (0.55, 0.91)*	0.71 (0.55, 0.91)
Bulgaria	0.98 (0.74, 1.22)	**1.22 (1.06, 1.41)**	1.21 (1.06, 1.41)
Croatia	1.01 (0.73, 1.26)	**0.99 (0.81, 1.16)**	0.99 (0.81, 1.16)
Cyprus	1.20 (1.04, 1.41)	**1.19 (1.03, 1.38)**	1.19 (1.03, 1.38)
Czechia	1.09 (0.89, 1.30)	**1.55 (1.37, 1.80)**	1.55 (1.37, 1.81)
Denmark	1.07 (0.88, 1.31)	**1.07 (0.91, 1.29)**	1.07 (0.91, 1.29)
Finland	0.87 (0.65, 1.11)	**1.17 (1.01, 1.39)**	1.17 (1.02, 1.38)
France	0.92 (0.67, 1.17)	**1.19 (1.03, 1.41)**	1.19 (1.03, 1.41)
Germany	1.01 (0.67, 1.25)	1.07 (0.93, 1.25)	**1.07 (0.90, 1.25) -**
Greece	1.08 (0.85, 1.31)	**1.65 (1.49, 1.84)**	1.65 (1.49, 1.84)
Hungary–Ireland	1.13 (0.95, 1.32)	**1.28 (1.15, 1.44)**	1.28 (1.16, 1.44)
Ireland	1.28 (1.10, 1.46)	**1.38 (1.24, 1.55)**	1.38 (1.24, 1.55)
Italy	0.84 (0.58, 1.13)	**1.17 (1.03, 1.34)**	1.17 (1.03, 1.34)
Netherlands	1.18 (0.97, 1.39)	**1.44 (1.28, 1.64)**	1.43 (1.28, 1.62)
Poland	1.01 (0.67, 1.23)	**1.72 (1.57, 1.91)**	1.72 (1.56, 1.91)
Portugal	1.16 (0.97, 1.41)	**1.23 (1.08, 1.41)**	1.23 (1.08, 1.41)
Romania–Slovakia	0.99 (0.68, 1.21)	**1.06 (0.92, 1.27)**	1.06 (0.91, 1.27)
Slovakia	1.01 (0.76, 1.25)	**1.66 (1.47, 1.91)**	1.66 (1.47, 1.92)
Slovenia	0.96 (0.76, 0.21)	**0.93 (0.76, 1.13)**	0.93 (0.76, 1.13)
Spain	1.07 (0.86, 1.29)	**1.61 (1.48, 1.80)**	1.62 (1.48, 1.80)
Sweden	1.12 (0.94, 1.34)	**1.22 (1.05, 1.44)**	1.22 (1.05, 1.43)

The values in parenthesis are the 95% confidence bands for the estimates of the differencing parameter. In bold, the selected model for each country according to the deterministic terms. *, evidence of mean reversion at the 5% level. Column 4, + / − positive or negative trend

**Table 4:** Estimates of the differencing parameter: total unemployment (levels 5–8)

Country	No terms	An intercept	An intercept and a linear time trend
Austria	0.68 (0.48, 0.88)	0.61 (0.45, 0.81)*	0.61 (0.44, 0.81)
Belgium	0.66 (0.43, 0.88)	0.39 (0.24, 0.57)*	0.38 (0.23, 0.56)
Bulgaria	0.84 (0.63, 1.08)	**1.01 (0.83, 1.22)**	1.01 (0.85, 1.21)
Croatia	0.83 (0.61, 1.14)	0.75 (0.58, 0.99)*	0.75 (0.58, 0.99)
Cyprus	1.02 (0.83, 1.21)	**1.06 (0.92, 1.24)**	1.06 (0.92, 1.24)
Czechia	0.95 (0.76, 1.19)	**0.92 (0.76, 1.15)**	0.92 (0.76, 1.15)
Denmark	0.84 (0.62, 1.07)	0.73 (0.58, 0.93)*	0.74 (0.58, 0.93)
Finland	0.87 (0.67, 1.09)	**0.89 (0.74, 1.09)**	0.89 (0.74, 1.09)
France	0.93 (0.67, 1.13)	**0.99 (0.83, 1.21)**	0.99 (0.83, 1.21)
Germany	1.01 (0.72, 1.24)	0.95 (0.81, 1.14)	**0.94 (0.78, 1.14) -**
Greece	1.21 (1.01, 1.42)	**1.59 (1.44, 1.76)**	1.58 (1.44, 1.75)
Hungary–Ireland	0.90 (0.75, 1.07)	**0.87 (0.74, 1.02)**	0.87 (0.74, 1.02)
Ireland	1.19 (1.03, 1.38)	**1.20 (1.07, 1.36)**	1.20 (1.07, 1.36)
Italy	0.88 (0.66, 1.13)	**0.78 (0.63, 1.00)**	0.78 (0.63, 1.00)
Netherlands	1.13 (0.93, 1.36)	**1.23 (1.08, 1.42)**	1.23 (1.08, 1.41)
Poland	1.01 (0.77, 1.26)	**1.13 (0.95, 1.34)**	1.13 (0.95, 1.34)
Portugal	0.88 (0.69, 1.07)	**0.95 (0.81, 1.11)**	0.95 (0.81, 1.11)
Romania–Slovakia	0.85 (0.65, 1.11)	**0.85 (0.69, 1.08)**	0.85 (0.69, 1.08)
Slovakia	1.13 (0.94, 1.36)	**1.21 (1.05, 1.45)**	1.21 (1.05, 1.45)
Slovenia	0.75 (0.53, 0.95)	**0.93 (0.79, 1.10)**	0.93 (0.79, 1.10)
Spain	1.08 (0.87, 1.32)	**1.53 (1.39, 1.71)**	1.53 (1.39, 1.71)
Sweden	0.89 (0.68, 1.08)	**0.84 (0.66, 1.06)**	0.84 (0.66, 1.06)

The values in parenthesis are the 95% confidence bands for the estimates of the differencing parameter. In bold, the selected model for each country according to the deterministic terms. *, evidence of mean reversion at the 5% level. Column 4, + / − positive or negative trend

**Table 5:** Degrees of persistence based on education level: total unemployment

Country	Low (0–2)	Medium (3–4)	High (5–8)
Austria	0.67 (0.52, 0.88)*	1.01 (0.83, 1.24)	0.61 (0.45, 0.81)*
Belgium	0.47 (0.34, 0.63)*	0.71 (0.55, 0.91)*	0.39 (0.24, 0.57)*
Bulgaria	1.20 (1.07, 1.37)	1.22 (1.06, 1.41)	1.01 (0.83, 1.22)
Croatia	0.93 (0.76, 1.16)	0.99 (0.81, 1.16)	0.75 (0.58, 0.99)*
Cyprus	1.02 (0.87, 1.20)	1.19 (1.03, 1.38)	1.06 (0.92, 1.24)
Czechia	1.30 (1.10, 1.59)	1.55 (1.37, 1.80)	0.92 (0.76, 1.15)
Denmark	0.87 (0.73, 1.06)	1.07 (0.91, 1.29)	0.73 (0.58, 0.93)*
Finland	0.56 (0.44, 0.72)*	1.17 (1.01, 1.39)	0.89 (0.74, 1.09)
France	0.96 (0.80, 1.17)	1.19 (1.03, 1.41)	0.99 (0.83, 1.21)
Germany	1.07 (0.90, 1.25) -	1.07 (0.90, 1.25) -	0.94 (0.78, 1.14) -
Greece	1.35 (1.21, 1.52)	1.65 (1.49, 1.84)	1.59 (1.44, 1.76)
Hungary–Ireland	1.15 (1.03, 1.29)	1.28 (1.15, 1.44)	0.87 (0.74, 1.02)
Ireland	1.29 (1.15, 1.45)	1.38 (1.24, 1.55)	1.20 (1.07, 1.36)
Italy	1.09 (0.95, 1.25)	1.17 (1.03, 1.34)	0.78 (0.63, 1.00)
Netherlands	1.28 (1.12, 1.50)	1.44 (1.28, 1.64)	1.23 (1.08, 1.42)
Poland	1.08 (0.92, 1.29)	1.72 (1.57, 1.91)	1.13 (0.95, 1.34)
Portugal	1.38 (1.25, 1.58)	1.23 (1.08, 1.41)	0.95 (0.81, 1.11)
Romania–Slovakia	0.73 (0.47, 1.09)	1.06 (0.92, 1.27)	0.85 (0.69, 1.08)
Slovakia	0.93 (0.75, 1.24)	1.66 (1.47, 1.91)	1.21 (1.05, 1.45)
Slovenia	0.79 (0.65, 0.95)*	0.93 (0.76, 1.13)	0.93 (0.79, 1.10)
Spain	1.86 (1.69, 2.08)	1.61 (1.48, 1.80)	1.53 (1.39, 1.71)
Sweden	0.85 (0.66, 1.10) +	1.22 (1.05, 1.44)	0.84 (0.66, 1.06)

The values in parenthesis are the 95% confidence bands for the estimates of the differencing parameter. In bold, the selected model for each country according to the deterministic terms. *, evidence of mean reversion at the 5% level. Column 4, + / − positive or negative trend

In general, we observe higher degrees of integration at the medium education level compared with low and high levels. In fact, the highest values of the differencing parameter *d* are observed at medium education level in all countries except for Spain and Portugal. For these two countries, the highest order of integration takes place at low education level followed by medium and then high level of education. For Belgium, the mean reversion (*d* < *1*) occurs at all education levels. Thus, for this country, shocks in unemployment rates are expected to be transitory, not requiring strong measures to recover the original long term projections. Though it is not in all cases, in many of them, the lowest are observed at the high education level (14 out of 22 countries).

In Tables 6, 7 and 8, we display the estimated orders of integration for the case of male unemployment for the different levels of education attainment. Table 9 contains a summary. Hence, from these tables, we find that, as with total unemployment series in general, we observe higher degrees of integration at the medium education level compared with low and high levels. More evidence of mean reversion for high levels of education and in general the lowest *d* occurring for this group of individuals. Thus, for 16 countries, the lowest *d* are found for high education level, while for 6, it is in the low level of education. It is again found that Germany displays a significant negative trend for low and high educational levels, as well as Poland, and Sweden has a positive trend.

**Table 6:** Estimates of the differencing parameter: male unemployment (levels 0–2)

Country	No terms	An intercept	An intercept and a linear time trend
Austria	0.76 (0.59, 0.96)	0.64 (0.51, 0.82)*	0.61 (0.45, 0.82)
Belgium	0.67 (0.40, 0.88)	0.55 (0.42, 0.69)*	0.54 (0.39, 0.69)
Bulgaria	0.96 (0.73, 1.18)	**1.31 (1.17, 1.49)**	1.31 (1.17, 1.49)
Croatia	0.81 (0.60, 1.05)	**0.79 (0.62, 1.02)**	0.79 (0.62, 1.02)
Cyprus	0.99 (0.83, 1.19)	**1.00 (0.85, 1.18)**	1.00 (0.85, 1.18)
Czechia	1.03 (0.84, 1.25)	**1.05 (0.90, 1.26)**	1.05 (0.90, 1.26)
Denmark	0.88 (0.67, 1.12)	**0.84 (0.70, 1.03)**	0.84 (0.70, 1.03)
Finland	0.92 (0.72, 1.20)	0.56 (0.40, 0.77)*	0.55 (0.38, 0.77)
France	0.90 (0.64, 1.14)	**1.00 (0.85, 1.17)**	1.00 (0.84, 1.17)
Germany	0.97 (0.65, 1.16)	0.91 (0.79, 1.07)	**0.88 (0.76, 1.07) -**
Greece	1.31 (1.14, 1.50)	**1.41 (1.27, 1.58)**	1.41 (1.27, 1.58)
Hungary–Ireland	0.98 (0.77, 1.15)	**1.06 (0.94, 1.19)**	1.06 (0.94, 1.19)
Ireland	1.23 (1.05, 1.44)	**1.26 (1.12, 1.44)**	1.26 (1.12, 1.43)
Italy	0.81 (0.58, 1.05)	**1.11 (0.94, 1.27)**	1.11 (0.94, 1.27)
Netherlands	1.13 (0.94, 1.26)	**1.24 (1.09, 1.43)**	1.24 (1.09, 1.42)
Poland	0.97 (0.68, 1.21)	1.01 (0.86, 1.20)	**1.01 (0.86, 1.20) -**
Portugal	1.19 (1.02, 1.38)	**1.36 (1.23, 1.53)**	1.36 (1.23, 1.52)
Romania–Slovakia	0.97 (0.79, 1.20)	**0.73 (0.49, 1.19)**	0.72 (0.42, 1.18)
Slovakia	0.97 (0.74, 1.19)	**0.93 (0.77, 1.18)**	0.93 (0.74, 1.18)
Slovenia	0.76 (0.59, 0.95)	0.69 (0.55, 0.84)*	0.69 (0.55, 0.84)
Spain	1.29 (1.11, 1.49)	**1.75 (1.60, 1.97)**	1.75 (1.60, 1.97)
Sweden	1.04 (0.85, 1.27)	0.84 (0.65, 1.10)	**0.89 (0.72, 1.09) + **

The values in parenthesis are the 95% confidence bands for the estimates of the differencing parameter. In bold, the selected model for each country according to the deterministic terms. *, evidence of mean reversion at the 5% level. Column 4, + / − positive or negative trend

**Table 7:** Estimates of the differencing parameter: male unemployment (levels 3–4)

Country	No terms	An intercept	An intercept and a linear time trend
Austria	0.93 (0.71, 1.15)	**0.92 (0.75, 1.14)**	0.92 (0.75, 1.14)
Belgium	0.85 (0.62, 1.09)	0.67 (0.52, 0.86)*	0.67 (0.52, 0.86)
Bulgaria	0.99 (0.75, 1.24)	**1.26 (1.11, 1.44)**	1.26 (1.11, 1.43)
Croatia	1.01 (0.78, 1.24)	**1.02 (0.86, 1.22)**	1.02 (0.86, 1.22)
Cyprus	1.25 (1.09, 1.43)	**1.38 (1.24, 1.55)**	1.38 (1.24, 1.55)
Czechia	1.14 (0.97, 1.36)	**1.46 (1.27, 1.70)**	1.46 (1.27, 1.70)
Denmark	1.06 (0.88, 1.28)	**1.12 (0.97, 1.32)**	1.12 (0.97, 1.32)
Finland	0.85 (0.64, 1.07)	**1.22 (1.04, 1.49)**	1.22 (1.04, 1.48)
France	0.96 (0.73, 1.21)	**1.07 (0.92, 1.27)**	1.07 (0.92, 1.27)
Germany	1.00 (0.72, 1.25)	**1.12 (0.96, 1.33)**	1.12 (0.95, 1.34)
Greece	1.18 (0.98, 1.39)	**1.66 (1.51, 1.83)**	1.66 (1.51, 1.83)
Hungary–Ireland	1.13 (0.95, 1.31)	**1.27 (1.16, 1.41)**	1.27 (1.16, 1.41)
Ireland	1.37 (1.22, 1.57)	**1.48 (1.32, 1.69)**	1.48 (1.32, 1.69)
Italy	0.78 (0.54, 1.04)	**1.01 (0.85, 1.18)**	1.00 (0.84, 1.18)
Netherlands	1.14 (0.94, 1.33)	**1.28 (1.13, 1.44)**	1.27 (1.13, 1.43)
Poland	1.01 (0.68, 1.23)	**1.68 (1.53, 1.86)**	1.67 (1.52, 1.86)
Portugal	1.10 (0.91, 1.34)	**1.04 (0.90, 1.21)**	1.04 (0.90, 1.21)
Romania–Slovakia	0.96 (0.71, 1.21)	**1.07 (0.89, 1.35)**	1.07 (0.88, 1.35)
Slovakia	1.01 (0.78, 1.24)	**1.70 (1.53, 1.95)**	1.71 (1.53, 1.98)
Slovenia	0.82 (0.62, 1.02)	0.80 (0.64, 0.97)*	0.80 (0.64, 0.97)
Spain	1.21 (1.03, 1.41)	**1.67 (1.54, 1.83)**	1.67 (1.54, 1.83)
Sweden	1.13 (0.96, 1.35)	**1.22 (1.03, 1.46)**	1.22 (1.03, 1.44)

The values in parenthesis are the 95% confidence bands for the estimates of the differencing parameter. In bold, the selected model for each country according to the deterministic terms. *, evidence of mean reversion at the 5% level. Column 4, + / − positive or negative trend

**Table 8:** Estimates of the differencing parameter: male unemployment (levels 5–8)

Country	No terms	An intercept	An intercept and a linear time trend
Austria	0.70 (0.49, 0.92)	0.61 (0.46, 0.80)*	0.60 (0.44, 0.80)
Belgium	0.68 (0.40, 0.90)	0.35 (0.19, 0.53)*	0.35 (0.21, 0.53)
Bulgaria	0.81 (0.60, 1.06)	**0.81 (0.64, 1.03)**	0.82 (0.65, 1.03)
Croatia	0.69 (0.48, 0.94)	0.61 (0.45, 0.81)*	0.61 (0.45, 0.81)
Cyprus	0.88 (0.70, 1.09)	**0.88 (0.73, 1.07)**	0.88 (0.73, 1.07)
Czechia	0.89 (0.72, 1.09)	0.82 (0.69, 0.99)*	0.81 (0.68, 0.99)
Denmark	0.65 (0.45, 0.87)	0.55 (0.38, 0.75)*	0.55 (0.38, 0.75)
Finland	0.74 (0.51, 0.98)	0.69 (0.54, 0.89)*	0.69 (0.52, 0.89)
France	0.89 (0.70, 1.08)	**0.81 (0.64, 1.02)**	0.81 (0.64, 1.02)
Germany	1.01 (0.77, 1.22)	0.97 (0.83, 1.15)	**0.96 (0.80, 1.15) -**
Greece	1.34 (1.19, 1.54)	**1.51 (1.36, 1.72)**	1.51 (1.36, 1.72)
Hungary–Ireland	0.90 (0.76, 1.07)	**0.88 (0.75, 1.04)**	0.88 (0.75, 1.04)
Ireland	1.25 (1.10, 1.45)	**1.36 (1.22, 1.53)**	1.36 (1.22, 1.53)
Italy	0.77 (0.54, 1.03)	0.65 (0.53, 0.86)*	0.64 (0.48, 0.85)
Netherlands	1.09 (0.90, 1.32)	**1.14 (1.00, 1.33)**	1.14 (1.00, 1.33)
Poland	1.03 (0.83, 1.31)	0.90 (0.74, 1.13)	**0.89 (0.72, 1.13) -**
Portugal	0.96 (0.79, 1.16)	**0.95 (0.82, 1.10)**	0.95 (0.82, 1.10)
Romania–Slovakia	0.82 (0.61, 1.13)	**0.81 (0.64, 1.06)**	0.82 (0.65, 1.07)
Slovakia	1.13 (0.95, 1.38)	**1.11 (0.93, 1.38)**	1.11 (0.93, 1.38)
Slovenia	0.49 (0.29, 0.71)	0.63 (0.48, 0.83)*	0.64 (0.47, 0.84)
Spain	1.11 (0.91, 1.34)	**1.45 (1.31, 1.62)**	1.45 (1.31, 1.62)
Sweden	0.84 (0.63, 1.03)	**0.78 (0.58, 1.03)**	0.79 (0.60, 1.03)

The values in parenthesis are the 95% confidence bands for the estimates of the differencing parameter. In bold, the selected model for each country according to the deterministic terms. In red and *, evidence of mean reversion at the 5% level. Column 4, + / − positive or negative trend

**Table 9:** Degrees of persistence based on education level: male unemployment

Country	Low (0–2)	Medium (3–4)	High (5–8)
Austria	0.64 (0.51, 0.82)*	0.92 (0.75, 1.14)	0.61 (0.46, 0.80)*
Belgium	0.55 (0.42, 0.69)*	0.67 (0.52, 0.86)*	0.35 (0.19, 0.53)*
Bulgaria	1.31 (1.17, 1.49)	1.26 (1.11, 1.44)	0.81 (0.64, 1.03)
Croatia	0.79 (0.62, 1.02)	1.02 (0.86, 1.22)	0.61 (0.45, 0.81)*
Cyprus	1.00 (0.85, 1.18)	1.38 (1.24, 1.55)	0.88 (0.73, 1.07)
Czechia	1.05 (0.90, 1.26)	1.46 (1.27, 1.70)	0.82 (0.69, 0.99)*
Denmark	0.84 (0.70, 1.03)	1.12 (0.97, 1.32)	0.55 (0.38, 0.75)*
Finland	0.56 (0.40, 0.77)*	1.22 (1.04, 1.49)	0.69 (0.54, 0.89)*
France	1.00 (0.85, 1.17)	1.07 (0.92, 1.27)	0.81 (0.64, 1.02)
Germany	0.88 (0.76, 1.07) -	1.12 (0.96, 1.33)	0.96 (0.80, 1.15) -
Greece	1.41 (1.27, 1.58)	1.66 (1.51, 1.83)	1.51 (1.36, 1.72)
Hungary–Ireland	1.06 (0.94, 1.19)	1.27 (1.16, 1.41)	0.88 (0.75, 1.04)
Ireland	1.26 (1.12, 1.44)	1.48 (1.32, 1.69)	1.36 (1.22, 1.53)
Italy	1.11 (0.94, 1.27)	1.01 (0.85, 1.18)	0.65 (0.53, 0.86)*
Netherlands	1.24 (1.09, 1.43)	1.28 (1.13, 1.44)	1.14 (1.00, 1.33)
Poland	1.01 (0.86, 1.20) -	1.68 (1.53, 1.86)	0.89 (0.72, 1.13) -
Portugal	1.36 (1.23, 1.53)	1.04 (0.90, 1.21)	0.95 (0.82, 1.10)
Romania–Slovakia	0.73 (0.49, 1.19)	1.07 (0.89, 1.35)	0.81 (0.64, 1.06)
Slovakia	0.93 (0.77, 1.18)	1.70 (1.53, 1.95)	1.11 (0.93, 1.38)
Slovenia	0.69 (0.55, 0.84)*	0.80 (0.64, 0.97)*	0.63 (0.48, 0.83)*
Spain	1.75 (1.60, 1.97)	1.67 (1.54, 1.83)	1.45 (1.31, 1.62)
Sweden	0.89 (0.72, 1.09) +	1.22 (1.03, 1.46)	0.78 (0.58, 1.03)

The values in parenthesis are the 95% confidence bands for the estimates of the differencing parameter. In bold, the selected model for each country according to the deterministic terms. *, evidence of mean reversion at the 5% level. Column 4, + / − positive or negative trend

In Tables 10, 11 and 12, we present the results for the case of female unemployment along with a summary of the results in Table 13.

**Table 10:** Estimates of the differencing parameter: female unemployment (levels 0–2)

Country	No terms	An intercept	An intercept and a linear time trend
Austria	0.86 (0.62, 1.09)	**0.51 (0.33, 0.79)**	0.52 (0.33, 0.80)
Belgium	0.82 (0.61, 1.03)	**0.35 (0.21, 0.54)**	0.31 (0.16, 0.52)
Bulgaria	0.93 (0.73, 1.17)	**0.97 (0.83, 1.13)**	0.97 (0.83, 1.13)
Croatia	0.85 (0.65, 1.11)	**0.91 (0.75, 1.12)**	0.91 (0.75, 1.12)
Cyprus	0.72 (0.53, 0.95)	**0.79 (0.64, 0.99)**	0.78 (0.62, 0.99)
Czechia	0.98 (0.78, 1.19)	**1.00 (0.84, 1.21)**	1.00 (0.83, 1.21)
Denmark	0.74 (0.52, 0.99)	**0.63 (0.47, 0.83)**	0.63 (0.46, 0.83)
Finland	0.74 (0.54, 0.95)	0.45 (0.33, 0.58)	**0.38 (0.23, 0.55) + **
France	0.82 (0.56, 1.04)	0.68 (0.55, 0.88)	**0.65 (0.48, 0.87) + **
Germany	1.01 (0.69, 1.25)	0.92 (0.78, 1.08)	**0.90 (0.72, 1.08) -**
Greece	0.96 (0.75, 1.19)	**1.18 (1.03, 1.35)**	1.18 (1.02, 1.35)
Hungary–Ireland	1.00 (0.80, 1.19)	**1.08 (0.96, 1.23)**	1.08 (0.96, 1.23)
Ireland	0.95 (0.79, 1.12)	**0.92 (0.78, 1.08)**	0.93 (0.79, 1.08)
Italy	0.96 (0.63, 1.11)	**0.96 (0.83, 1.13)**	0.96 (0.82, 1.13)
Netherlands	0.97 (0.78, 1.18)	**1.05 (0.90, 1.23)**	1.05 (0.90, 1.23)
Poland	0.93 (0.71, 1.16)	0.91 (0.73, 1.15)	**0.91 (0.72, 1.15) -**
Portugal	0.95 (0.74, 1.16)	**1.07 (0.94, 1.22)**	1.07 (0.94, 1.22)
Romania–Slovakia	0.85 (0.64, 1.08)	**0.64 (0.43, 0.92)**	0.64 (0.43, 0.92)
Slovakia	1.00 (0.80, 1.22)	0.82 (0.65, 1.20)	**0.78 (0.50, 1.20) -**
Slovenia	0.59 (0.41, 0.80)	**0.71 (0.55, 0.93)**	0.71 (0.55, 0.93)
Spain	1.00 (0.78, 1.26)	**1.70 (1.54, 1.91)**	1.70 (1.55, 1.92)
Sweden	0.90 (0.72, 1.10)	0.60 (0.47, 0.84)	**0.65 (0.44, 0.88) + **

The values in parenthesis are the 95% confidence bands for the estimates of the differencing parameter. In bold, the selected model for each country according to the deterministic terms. *, evidence of mean reversion at the 5% level. Column 4, + / − positive or negative trend

**Table 11:** Estimates of the differencing parameter: female unemployment (levels 3–4)

Country	No terms	An intercept	An intercept and a linear time trend
Austria	0.83 (0.58, 1.04)	**0.67 (0.51, 0.87)**	0.67 (0.51, 0.87)
Belgium	0.83 (0.60, 1.04)	0.50 (0.36, 0.67)	**0.40 (0.23, 0.62) -**
Bulgaria	0.93 (0.65, 1.18)	0.98 (0.82, 1.19)	**0.97 (0.83, 1.18) -**
Croatia	0.86 (0.58, 1.13)	0.71 (0.54, 0.89)	**0.71 (0.54, 0.89) -**
Cyprus	0.96 (0.78, 1.17)	**0.89 (0.71, 1.09)**	0.89 (0.72, 1.09)
Czechia	1.03 (0.81, 1.25)	**1.45 (1.27, 1.68)**	1.45 (1.27, 1.68)
Denmark	0.90 (0.67, 1.14)	**0.73 (0.55, 0.93)**	0.73 (0.55, 0.93)
Finland	0.88 (0.64, 1.14)	**0.74 (0.58, 0.93)**	0.75 (0.60, 0.93)
France	0.89 (0.59, 1.11)	**0.96 (0.81, 1.17)**	0.96 (0.80, 1.17)
Germany	1.02 (0.66, 1.25)	0.95 (0.83, 1.11)	**0.92 (0.74, 1.11) -**
Greece	1.01 (0.77, 1.25)	**1.51 (1.35, 1.70)**	1.51 (1.35, 1.70)
Hungary–Ireland	1.08 (0.89, 1.27)	**1.14 (1.01, 1.30)**	1.14 (1.01, 1.30)
Ireland	1.00 (0.77, 1.19)	**1.02 (0.88, 1.16)**	1.02 (0.88, 1.16)
Italy	0.86 (0.58, 1.15)	**1.17 (1.04, 1.34)**	1.17 (1.04, 1.34)
Netherlands	1.12 (0.93, 1.33)	**1.31 (1.16, 1.49)**	1.31 (1.16, 1.49)
Poland	1.00 (0.68, 1.23)	**1.62 (1.45, 1.83)**	1.62 (1.44, 1.83)
Portugal	1.04 (0.86, 1.26)	**1.11 (0.96, 1.28)**	1.11 (0.96, 1.28)
Romania–Slovakia	0.89 (0.58, 1.11)	0.70 (0.52, 0.91)	**0.70 (0.53, 0.91) -**
Slovakia	1.01 (0.75, 1.25)	**1.48 (1.30, 1.75)**	1.48 (1.30, 1.75)
Slovenia	0.92 (0.71, 1.18)	**0.79 (0.62, 1.01)**	0.78 (0.62, 1.00)
Spain	0.97 (0.74, 1.20)	**1.39 (1.25, 1.56)**	1.39 (1.25, 1.56)
Sweden	1.03 (0.84, 1.24)	**1.00 (0.84, 1.19)**	1.00 (0.84, 1.19)

The values in parenthesis are the 95% confidence bands for the estimates of the differencing parameter. In bold, the selected model for each country according to the deterministic terms. *, evidence of mean reversion at the 5% level. Column 4, + / − positive or negative trend

**Table 12:** Estimates of the differencing parameter: female unemployment (levels 5–8)

Country	No terms	An intercept	An intercept and a linear time trend
Austria	0.41 (0.01, 0.66)	**0.19 (0.01, 0.45)**	0.19 (0.00, 0.45)
Belgium	0.59 (0.38, 0.81)	0.34 (0.16, 0.56)	**0.32 (0.15, 0.55) -**
Bulgaria	0.83 (0.63, 1.07)	**0.99 (0.81, 1.20)**	0.99 (0.84, 1.19)
Croatia	0.59 (0.36, 0.86)	**0.47 (0.31, 0.68)**	0.47 (0.32, 0.68)
Cyprus	1.01 (0.83, 1.19)	**1.09 (0.96, 1.25)**	1.09 (0.96, 1.25)
Czechia	0.83 (0.65, 1.05)	**0.79 (0.65, 0.98)**	0.79 (0.65, 0.98)
Denmark	0.90 (0.71, 1.08)	**0.69 (0.54, 0.87)**	0.69 (0.55, 0.87)
Finland	0.85 (0.66, 1.05)	**0.75 (0.61, 0.93)**	0.75 (0.61, 0.93)
France	0.92 (0.64, 1.14)	**0.84 (0.69, 1.00)**	0.84 (0.70, 1.00)
Germany	0.97 (0.66, 1.18)	**0.92 (0.74, 1.11) -**	**0.69 (0.56, 0.84) -**
Greece	1.11 (0.89, 1.34)	**1.56 (1.43, 1.72)**	1.56 (1.42, 1.71)
Hungary–Ireland	0.88 (0.72, 1.05)	**0.81 (0.68, 0.98)**	0.81 (0.68, 0.97)
Ireland	102 (0.85, 1.20)	**0.98 (0.85, 1.13)**	0.98 (0.85, 1.13)
Italy	0.81 (0.58, 1.05)	**0.60 (0.45, 0.80)**	0.61 (0.45, 0.80)
Netherlands	1.12 (0.94, 1.33)	**1.19 (1.02, 1.39)**	1.19 (1.02, 1.38)
Poland	0.97 (0.74, 1.21)	**1.18 (1.01, 1.39)**	1.18 (1.02, 1.39)
Portugal	0.79 (0.61, 0.99)	**0.82 (0.68, 0.99)**	0.82 (0.68, 0.99)
Romania–Slovakia	0.81 (0.62, 1.04)	**0.74 (0.58, 0.95)**	0.73 (0.57, 0.95)
Slovakia	1.05 (0.86, 1.28)	**1.13 (0.99, 1.32)**	1.13 (0.99, 1.32)
Slovenia	0.89 (0.70, 1.09)	**0.96 (0.81, 1.12)**	0.96 (0.81, 1.12)
Spain	1.05 (0.83, 1.29)	**1.54 (1.38, 1.73)**	1.54 (1.38, 1.75)
Sweden	0.86 (0.66, 1.05)	**0.71 (0.54, 0.92)**	0.72 (0.55, 0.92)

The values in parenthesis are the 95% confidence bands for the estimates of the differencing parameter. In bold, the selected model for each country according to the deterministic terms. *, evidence of mean reversion at the 5% level. Column 4, + / − positive or negative trend

**Table 13:** Degrees of persistence based on education level: female unemployment

Country	Low (0–2)	Medium (3–4)	High (5–8)
Austria	0.51 (0.33, 0.79)*	0.67 (0.51, 0.87)*	0.19 (0.01, 0.45)*
Belgium	0.35 (0.21, 0.54)*	0.40 (0.23, 0.62)* -	0.32 (0.15, 0.55)* -
Bulgaria	0.97 (0.83, 1.13)	0.97 (0.83, 1.18) -	0.99 (0.81, 1.20)
Croatia	0.91 (0.75, 1.12)	0.71 (0.54, 0.89)* -	0.47 (0.31, 0.68)*
Cyprus	0.79 (0.64, 0.99)*	0.89 (0.71, 1.09)	1.09 (0.96, 1.25)
Czechia	1.00 (0.84, 1.21)	1.45 (1.27, 1.68)	0.79 (0.65, 0.98)*
Denmark	0.63 (0.47, 0.83)*	0.73 (0.55, 0.93)*	0.69 (0.54, 0.87)*
Finland	0.38 (0.23, 0.55) * +	0.74 (0.58, 0.93)*	0.75 (0.61, 0.93)*
France	0.65 (0.48, 0.87) * +	0.96 (0.81, 1.17)	0.84 (0.69, 1.00)*
Germany	0.90 (0.72, 1.08) -	0.95 (0.83, 1.11)	0.69 (0.56, 0.84)* -
Greece	1.18 (1.03, 1.35)	1.51 (1.35, 1.70)	1.56 (1.43, 1.72)
Hungary–Ireland	1.08 (0.96, 1.23)	1.14 (1.01, 1.30)	0.81 (0.68, 0.98)*
Ireland	0.92 (0.78, 1.08)	1.02 (0.88, 1.16)	0.98 (0.85, 1.13)
Italy	0.96 (0.83, 1.13)	1.17 (1.04, 1.34)	0.60 (0.45, 0.80)*
Netherlands	1.05 (0.90, 1.23)	1.31 (1.16, 1.49)	1.19 (1.02, 1.39)
Poland	0.91 (0.72, 1.15) -	1.62 (1.45, 1.83)	1.18 (1.01, 1.39)
Portugal	1.07 (0.94, 1.22)	1.11 (0.96, 1.28)	0.82 (0.68, 0.99)*
Romania–Slovakia	0.64 (0.43, 0.92)*	0.70 (0.53, 0.91)* -	0.74 (0.58, 0.95)*
Slovakia	0.78 (0.50, 1.20) -	1.48 (1.30, 1.75)	1.13 (0.99, 1.32)
Slovenia	0.71 (0.55, 0.93)	0.79 (0.62, 1.01)	0.96 (0.81, 1.12)
Spain	1.70 (1.54, 1.91)	1.39 (1.25, 1.56)	1.54 (1.38, 1.73)
Sweden	0.65 (0.44, 0.88) * +	1.00 (0.84, 1.19)	0.71 (0.54, 0.92)*

The values in parenthesis are the 95% confidence bands for the estimates of the differencing parameter. In bold, the selected model for each country according to the deterministic terms. *, evidence of mean reversion at the 5% level. Column 4, + / − positive or negative trend

From these tables, we find that at low levels of education, there are significant positive trends in Finland, France and Sweden, while negative ones for Germany, Poland and Slovakia. At the medium level, the five significant trends are negative (Belgium, Bulgaria, Croatia, Germany and Romania). For high levels of education, only two negative trends are detected: Belgium and Germany. In all the other cases, the time trend coefficients are found to be statistically insignificantly different from zero. As with the total unemployment series, in general, we observe higher degrees of integration at the medium education level compared with low and high levels. For 14 countries, the highest value of *d* is found for the medium level of education. For six countries (Bulgaria, Cyprus, Finland, Greece, Romania and Slovenia), the highest *d* occurs at high level of education, while for Croatia and Spain, this happens at the low level. Also, more evidence of mean reversion (*d* < 1) and thus transitory shocks is found in high levels of education (Table 14).

**Table 14:** Summary results: Averaged values

	Low educ	Medium educ	High educ
Total	1.037 (0.885, 1.244)	1.251 (1.091, 1.451)	0.972 (0.818, 1.171)
Male	1.002 (9.696, 1.119)	1.232 (1.075, 1.445)	0.875 (0.741, 1.051)
Female	0.851 (0.687, 1.060)	1.032 (0.875, 1.226)	0.865 (0.714, 1.054)

## Conclusion

In this paper, we have examined the unemployment rate series corresponding to a group of 22 European countries and for the time period from 2003Q2 to 2019Q4. Using fractional integration methods, we have tried to determine if shocks in the series of unemployment disaggregated by sex and education have transitory or permanent effects.

Our results indicate high levels of persistence in all cases, with values close to 1 in the majority of the series. In general, higher degrees of integration are observed in unemployed males than in females, and looking at the level of schooling, the highest degree of persistence is obtained at the medium education level. In addition, it is also observed that females display lower degrees of integration than males; however, for females, a lower value for *d* is obtained at the low education level. These results indicate that in the event of shocks affecting unemployment rates, long-lasting effects are observed in all cases, as well as higher degrees of dependence in males and at medium education levels.

This article can be extended in various directions. Thus, for example, structural breaks can be taken into account. There are exit procedures for testing *I*(*d*) models in the context of known and unknown breaks (Gil-Alana, 2004, 2008; Sibbersten, 2004; Choi et al., 2010; etc.); however, structural breaks produce abrupt changes in the data, and they can be avoided by using non-linear deterministic trends like those proposed in Cuestas and Gil-Alana (2016) (and based on Chebyshev polynomials in time) or using Fourier functions in time (Gil-Alana & Yaya, 2021) or neural networks (Yaya et al., 2021). Works in these directions are now in progress.

## Data Availability

The data is available at Eurostat (https://ec.europa.eu/eurostat). The code of the variable is une_educ_q.

## Footnotes

1 See also Ghoshray et al. (2016).
